# Efficacy of machine learning in simulating precipitation and its extremes over the capital cities in North Indian states

**DOI:** 10.1038/s41598-024-84360-w

**Published:** 2025-03-25

**Authors:** Aayushi Tandon, Amit Awasthi, Kanhu Charan Pattnayak

**Affiliations:** 1 Department of Applied Sciences, University of Petroleum & Energy Studies, Dehradun, Uttarakhand India; 2 School of Earth and Environment, University of Leeds, Leeds, UK; 3 Cambridge Judge Business School, Cambridge University, Cambridge, UK

**Keywords:** Climate change, Machine learning, North Indian states, Precipitation patterns, Random forest, Support vector machine, Hydrology, Atmospheric dynamics, Climate and Earth system modelling

## Abstract

Climate change-induced precipitation extremes are a pressing global concern. This study investigates the predictability of precipitation patterns and extremes across North Indian states from 1984 to 2023 using NASA’s Prediction of Worldwide Energy Resources (POWER) datasets and machine learning (ML) models. The current ML model builds on the relationship between rainfall and key climatic parameters such as dew point temperature and relative humidity, showing a strong positive correlation (CC = 0.4) significant at the 0.05 level. In simulating precipitation, Random Forest Classifier (RFC) achieved the highest accuracy (~ 83%) for Rajasthan and Uttar Pradesh, while Support Vector Classifier (SVC) performed best (79–83% accuracy) in other states. However, ML models exhibited approximately 5% lower skill in higher elevated stations as compared to lower ones, due to differing atmospheric mechanisms. For extreme precipitation events (10th and 95th percentiles of intensity), RFC consistently outperformed SVC across all states showing superior ability to distinguish extreme from non-extreme events (Area Under Curve ~ 0.90) and better model calibration (Brier Scores ~ 0.01). The developed ML models effectively simulated precipitation and extreme patterns, with RFC excelling at classifying extreme events. These findings can aid disaster preparedness and water resource management in regions with varied topography and complex terrain.

## Introduction

Extreme precipitation events, intensified by global warming, have become significant challenges for both society and the environment. These events, characterized by intense precipitation, can inflict severe damage on agriculture, ecosystems, and human communities^1–5^. As atmospheric moisture content rises due to climate change, these events are projected to become more frequent and intense. Observational data since the 1950s indicate a noticeable increase in the occurrence of heavy precipitation events in many regions, a trend expected to continue, according to the Intergovernmental Panel on Climate Change^6^. The sixth assessment report (AR6) also underscores that local communities, particularly those with limited adaptive capacity, are disproportionately vulnerable to these impacts^7^.

The mechanisms associated with the extreme precipitation events during the monsoon season, mostly triggered by the interactions between westward-moving monsoon systems, eastward-moving mid-tropospheric westerly troughs, and the rugged Himalayan topography, have had devastating consequences in various parts of Uttarakhand, Himachal Pradesh, and Jammu & Kashmir. Notable incidents include the Kedarnath tragedy (2013)^8,9^ and Uttarakhand cloudburst (2022, 2017, 2012)^10–12^, Lahaul-Spiti (July 2021), Mandi (July 2015)^13,14^, Leh cloudburst (2015, 2010)^15,16^, and the Jammu & Kashmir floods (2015, Sonmarg, Pahalgam, Ganderbal and Baltal)^16^ and many more reported in detail in a study conducted by Dimri et al., (2017). These events, often caused by the interaction of multiple atmospheric dynamics, lead to excessive precipitation, casualties, and significant infrastructural damage.

In North India, extreme precipitation events result from complex dynamics involving both large-scale atmospheric influences and localized factors. The unique topography of North India, particularly the Himalayas, plays a crucial role in shaping the Indian monsoon^17–21^. The interplay between the mountainous terrain and atmospheric disturbances, coupled with cold air intrusion from northern latitudes, creates conditions conducive to extreme precipitation events, as seen in Jammu and Kashmir during January 2017 ^22^. Western disturbances, embedded in the eastward-moving upper tropospheric Rossby wave train, contribute significantly to heavy precipitation in the Western Himalayas, especially during winter^23–25^. The high temperatures over the mountains and neighbouring areas contribute to the formation of low-pressure systems, which extend southward across the plains of South Asia, facilitating the northward progression of the monsoon^26–28^. The 2013 Uttarakhand disaster, which involved rapid monsoon progression and heavy precipitation, resulted from a combination of these large-scale circulations, orographic lifting, and intense convective activity^29^. These extreme precipitation events are known to exacerbate due to climate change with increased frequency and intensity^16,30^.

Traditional modeling approaches struggle to capture the complex interplay of factors shaping precipitation patterns, including climate change, topography, and atmospheric dynamics^31–33^. In response, integrating machine learning (ML) techniques has emerged as a promising avenue for understanding precipitation^34–37^ and extreme precipitation^38–41^ variability and enhancing forecasting accuracy leveraging large meteorological datasets and computational power to improve predictive capabilities amid climate change uncertainties^34–36^.

For India, a few literatures are available for extreme precipitation analysis^30,42–44^, however relatively fewer for extreme precipitation modelling using machine learning^37,45,46^. The limited availability of high-quality and comprehensive meteorological data in India and complexity of the regional topography and climate are also prominent contributing factor for limited number of research. A study by Ray et al. (2022) suggest that machine learning techniques, can effectively predict precipitation by correlating meteorological parameters with precipitation events^47^.

In light of aforesaid reasons, our study aims to explore key atmospheric variables affecting precipitation patterns and extreme events in North India and studies link between these variables and precipitation intensity across regions. This analysis provides a solid foundation for understanding the complex interplay between these variables and precipitation patterns.

Building upon this foundation, the study employs machine learning models to tackle the challenge of classifying precipitation events at different thresholds. These models are trained and evaluated on different data splits, with a primary objective of accurately determining whether precipitation will occur or not within a given timeframe and geographic area. The performance of these models is meticulously assessed using accuracy as a metric for decision making, ensuring model’s reliability and robustness. Recognizing the significance of extreme precipitation events, the study takes a step further by defining thresholds based on the 10th and 95th percentiles of precipitation intensity. These thresholds serve as benchmarks for identifying extreme event occurrences, both in terms of low and high precipitation levels. The machine learning models are then evaluated on their ability to accurately classify events falling within these extreme percentile ranges. Ultimately, the goal is to harness ML to improve the accuracy and reliability of precipitation forecasts, helping policymakers and communities prepare for and mitigate the impact of extreme weather events in the context of climate change.

## Study area

**Fig. 1:**
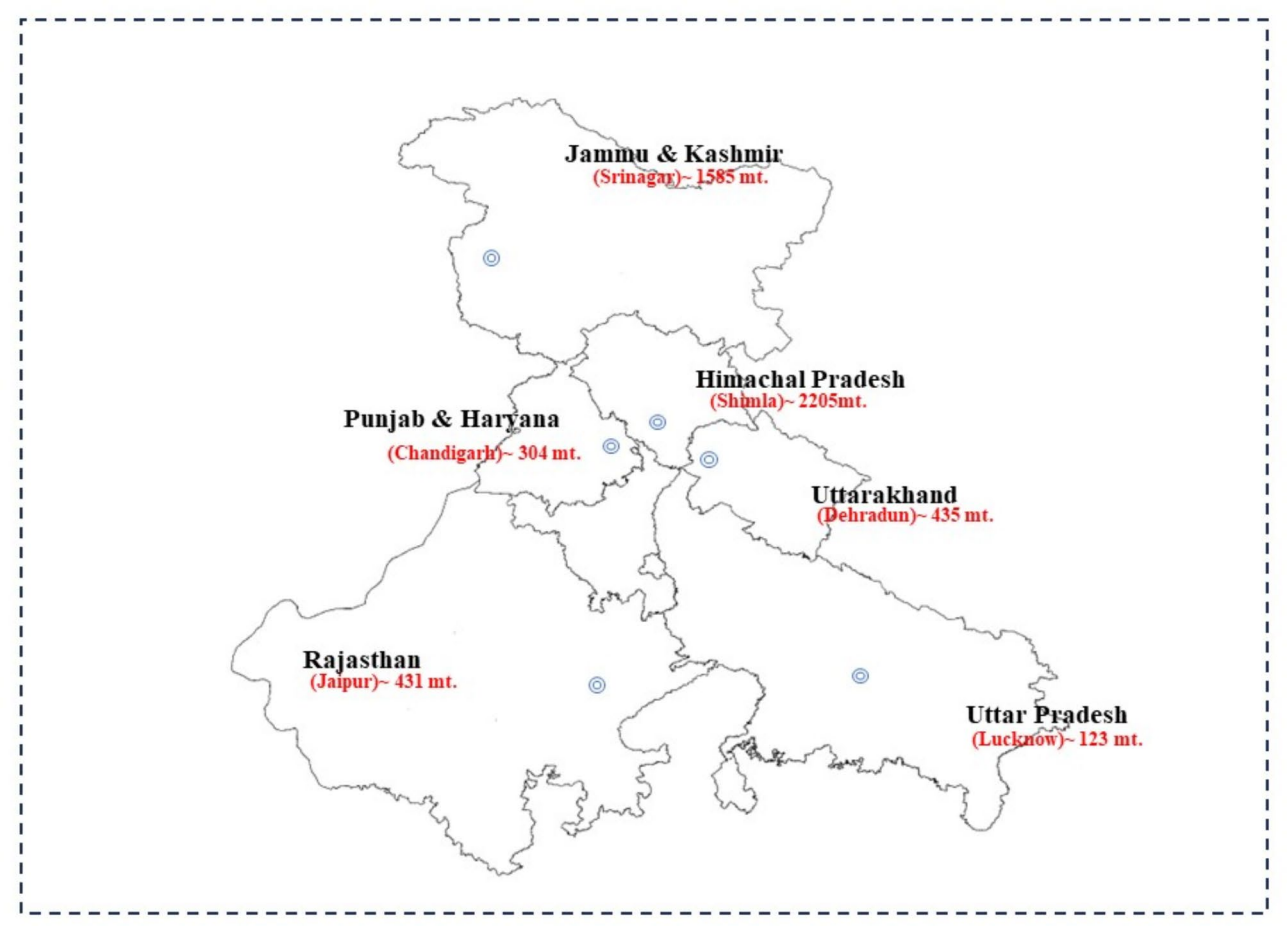
Study area map highlighting the geographical region under investigation. *(Source*: DIVA-GIS and ArcGIS^®^ software by Esri).

Encompassing a vast region in North India, this study area covers seven states: Himachal Pradesh (HP), Jammu and Kashmir (JK), Punjab and Haryana (PH) (considered together), Rajasthan (RJ), Uttarakhand (UK), and Uttar Pradesh (UP) (Fig. 1). The dramatic elevation range stretches from a low point of 60 m in Uttar Pradesh to a staggering 8,611 meters at K2 Peak in Jammu and Kashmir. This translates to significant temperature variations across the region. The plains experience scorching summers with highs reaching 45 °C, particularly in the Rajasthan’s Thar Desert, while winters can be mild with lows around 0 °C. In contrast, the hilly regions offer cooler summers with highs of 25 °C, but winters can be harsh with temperatures dipping as low as -30 °C in Jammu and Kashmir^48,49^.

Each state within this diverse landscape faces distinct environmental challenges^50^. The Himalayan states like Uttarakhand, Himachal Pradesh, and Jammu and Kashmir grapple with issues like forest fires, biodiversity loss, glacial retreat leading to water scarcity, and soil erosion. Meanwhile, the plains states of Uttar Pradesh, Punjab & Haryana, and Rajasthan battle water scarcity, soil degradation, and air pollution. Additionally, all states face challenges related to the impact of climate change on agriculture^51,52^. Understanding this interplay between natural processes and human activities across this vast region with its varying elevations and temperatures is crucial for developing effective management strategies and sustainable development practices^30,51^.

## Climatic condition and regional characteristics of precipitation in North India

North India’s climate is diverse due to its varied topography, which includes the Himalayas, the Thar Desert, and the Indo-Gangetic Plain^49,53–55^. The Himalayan region, encompassing Himachal Pradesh, Uttarakhand, Jammu & Kashmir, and Ladakh, experiences an alpine and subtropical climate with cold winters, mild summers, and heavy snowfall in higher altitudes^56,57^. The Indo-Gangetic Plain, which covers states like Punjab, Haryana, and Uttar Pradesh, has a subtropical monsoon climate characterized by hot summers, cold winters, and a distinct monsoon season from June to September^53,58,59^. The Thar Desert in Rajasthan features an arid climate with extreme temperatures and scanty rainfall^60,61^. These regions are influenced by the monsoon winds that bring significant rainfall to the plains and southern slopes of the Himalayas while creating a rain shadow effect in the desert areas^54,62^.

Seasonal variations play a significant role in shaping the climate across North India. Summers (April to June) are marked by extreme heat, particularly in the plains and desert areas, with temperatures often exceeding 45 °C^48^. The southwest monsoon season (June to September) brings heavy rains essential for agriculture but also poses risks of flooding and landslides in the Himalayan region^63^.

Autumn (October to November) is a transition period with receding monsoon rains and cooling temperatures. Winters (December to February) are cold, with western disturbances bringing rain and occasional snowfall to the plains and mountains. Spring (March to April) sees gradual warming and blooming flowers, marking the end of winter and the onset of the hot season. The Himalayas significantly influence these patterns by acting as a barrier to cold winds and affecting monsoon rainfall distribution^48^.

**Fig. 2:**
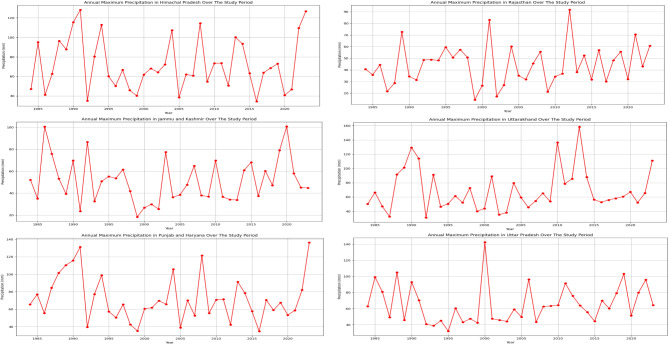
Annual maximum precipitation (mm), over the study period (1984–2023) for North Indian states.

The precipitation characteristics across the North Indian states exhibit significant variations, as evident from the analysis of annual maximum precipitation (Fig. 2), and threshold values corresponding to the 10th and 95th percentiles (Table 1). 

Uttarakhand (UK) experienced the highest maximum precipitation of 158.40 mm, followed by Punjab (PH) with 136.26 mm and Uttar Pradesh (UP) with 142.43 mm, representing regions with relatively higher precipitation levels. In contrast, Rajasthan (RJ) and Jammu and Kashmir (JK) recorded lower maximum precipitation values of 91.75 mm and 100.79 mm respectively, indicating drier conditions. Uttarakhand exhibited the highest standard deviation of 8.30 mm, suggesting greater variability in precipitation values around the maximum, while Rajasthan had the lowest standard deviation of 4.77 mm, indicating a more consistent pattern.

**Table 1:** Characteristics of precipitation across different states.

States	Maximum precipitation (mm)	Standard Deviation (mm)	10th percentile		95th percentile	
			Threshold (mm)	Event count [< threshold (day)]	Threshold (mm)	Event count [> threshold] (day)
HP	128.19	7.81	0.03	6631	23.74	363
JK	100.79	5.73	0.03	7104	17.71	387
PH	136.26	7.73	0.05	6118	24.23	344
RJ	91.75	4.77	0.03	5132	17.88	284
UK	158.40	8.30	0.04	6471	27.58	355
UP	142.43	7.48	0.04	5928	24.96	328

The 10th percentile thresholds, representing low precipitation events, ranged from 0.03 mm in Himachal Pradesh (HP), Jammu and Kashmir (JK), and Rajasthan (RJ) to 0.05 mm in Punjab (PH). The 95th percentile thresholds, indicating extreme high precipitation events, varied from 17.71 mm in Jammu and Kashmir to 27.58 mm in Uttarakhand. Jammu and Kashmir experienced the highest number of events (7104) below the 10th percentile threshold, while Himachal Pradesh recorded the highest (363) above the 95th percentile.

Overall, regional disparities exist, with higher maximum precipitation in Uttarakhand, Punjab, and Uttar Pradesh indicating wetter conditions, contrasted by drier conditions in Rajasthan and Jammu and Kashmir. The state of Uttarakhand exhibited greater precipitation variability, while Rajasthan had a more consistent pattern for precipitation during the study period. The state of Jammu and Kashmir had the highest frequency of low precipitation events, while Himachal Pradesh experienced more extreme high precipitation events. Meanwhile, the region-specific analysis and modeling approaches are crucial to capture the unique precipitation dynamics across the diverse North Indian states.

## Results

### State specific inter-variable analysis

**Fig. 3:**
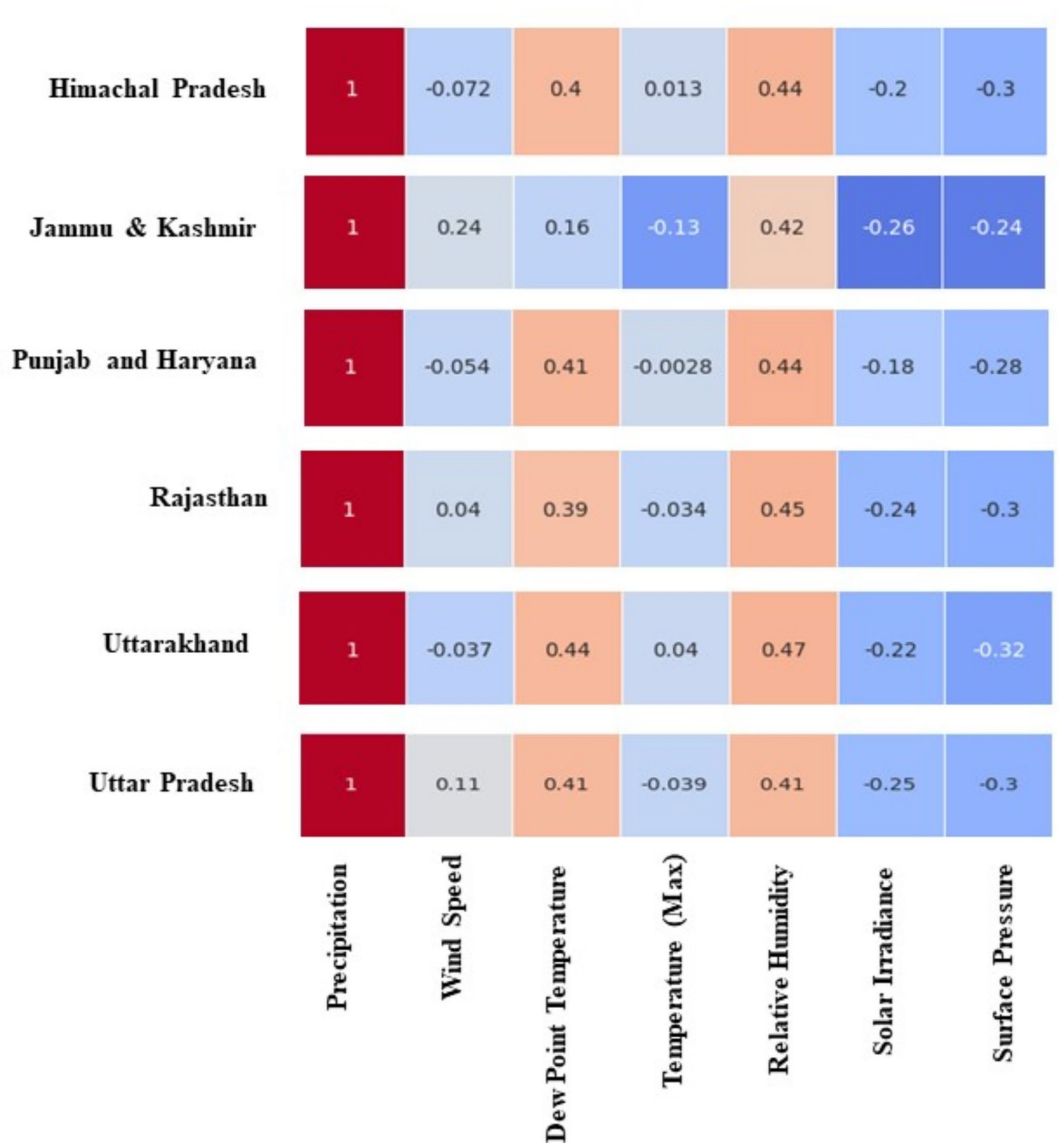
Matrix illustrating relationships between precipitation and other atmospheric variable for each of the states.

Understanding the intricate relationships between atmospheric variables is crucial for accurate classification of precipitation events^39^. In this section these intricacies are explored by examining state-specific inter-variable interactions. For each state’s capital included in the study, correlation matrices were generated (Fig. 3). These matrices provide a valuable tool to explore the strength and direction of linear associations between the chosen atmospheric variables (temperature, humidity, pressure, etc.). By analyzing these correlations, we aim to identify recurring patterns, potential dependencies between variables within each state, and any instances of multicollinearity.

Examining these state-specific relationships allows for a more nuanced understanding of how atmospheric variables interact and influence precipitation patterns across diverse geographical regions within North India^64^.

Across all states, a consistent theme emerges: Dew Point Temperature, Surface Pressure and Relative Humidity tend to be the strongest allies of precipitation. Strong Positive correlations of Dew Point Temperature, Surface Pressure (~ 0.4) and Strong negative correlations (~ -0.3) of Relative Humidity highlights their potential role as significant contributors to increased precipitation, when compared to all variables considered in this study. For states like HP and UK, precipitation exhibit positive correlations (0.013, 0.04) with temperature, while PH, JK, and UP show weaker negative (-0.0028 to -0.04) associations, pointing towards a more precise observation, while considering only temperature as a variable affecting precipitation over the state.

In almost all states, solar irradiance and surface pressure acts as a counterpoint to precipitation, exhibiting negative correlations ranging from − 0.18 in PH (Solar irradiance) to a more pronounced − 0.3 in HP, RJ, UK and UP (Surface pressure). The impact of wind on precipitation varies across states. While RJ, UP and JK show modest positive correlations (0.04, 0.11 and 0.24), other states like HP, PH and UK exhibit weakly negative associations (-0.072, -0.054 and 0.037) respectively with wind (Fig. 3).

Overall precipitation patterns across the study region are influenced by a complex interplay of various atmospheric factors, both directly and indirectly. In the direct category, elevated levels of dew point temperature, relative humidity and surface pressure emerge as significant contributors to increased precipitation in the study locations. These states consistently exhibit strong correlations between Dew Point Temperature, Surface Pressure, Relative Humidity, and precipitation, underscoring the direct impact of these meteorological variables on precipitation. Additionally, surface pressure and solar irradiance displays a notable indirect relationship, with lower level of the events associated with higher precipitation events across the study location, however correlation with surface pressure was only statistically significant at significance level of 0.05. Conversely, temperature and wind demonstrate a varied impact, with both positive and negative correlations observed in different states, indicating that warmer and windier conditions may contribute to precipitation in some regions while having the opposite effect in others (Fig. 3).

### ML model performance in precipitation classification

**Fig. 4:**
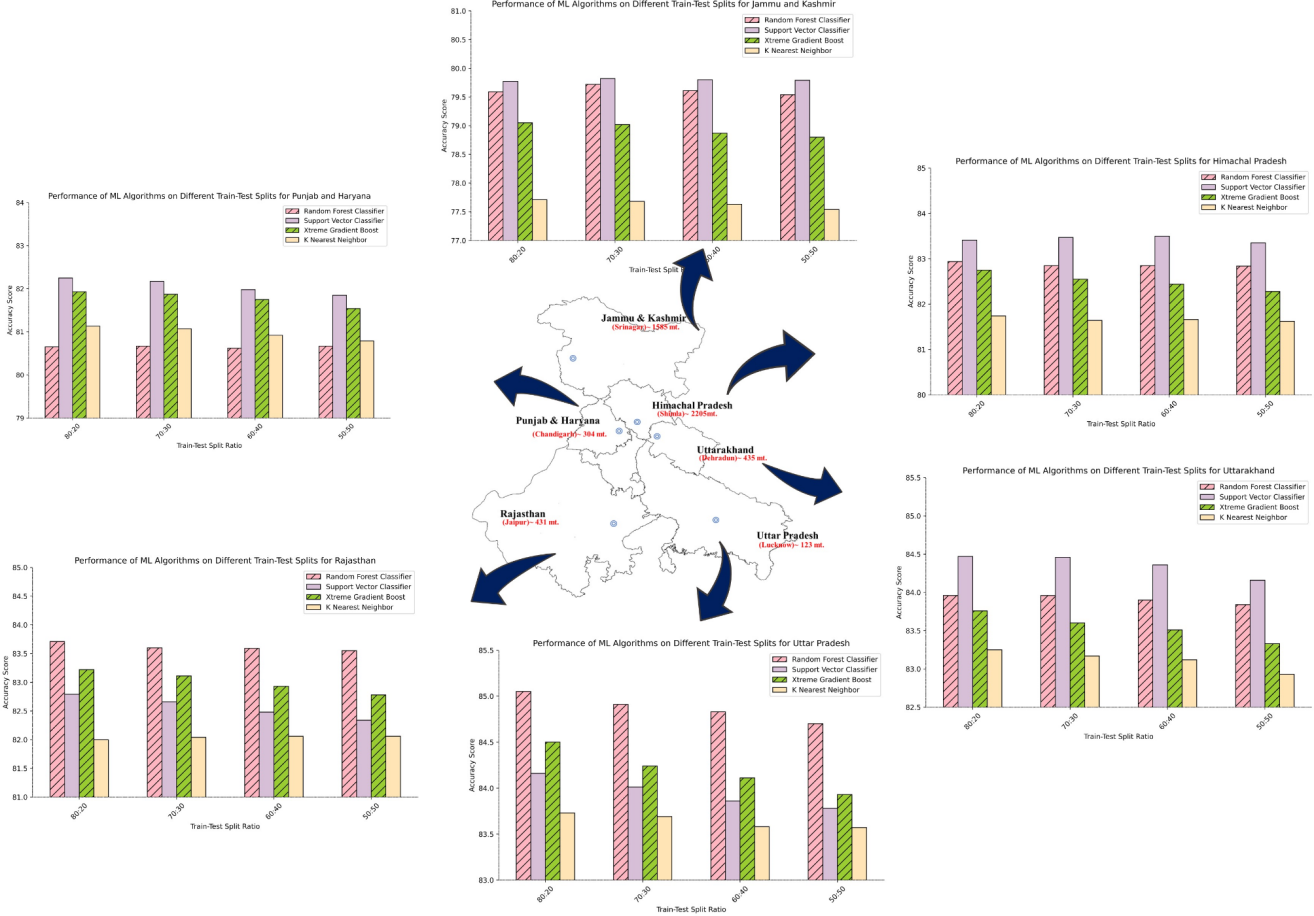
Machine learning accuracy across various train-test splits over North Indian states.

Taking these variables under consideration, four machine learning models, namely – Support Vector Classifier (SVC), Random Forest Classifier (RFC), XGBoost, and K Nearest Neighbors (KNN) – were developed to analyze precipitation and its extreme patterns over the study locations. The models were trained over the pre-processed dataset to achieve the highest possible accuracies. The developed model was trained to predict not only simple precipitation events, but extreme precipitation events at different threshold levels also. The developed model was trained and tested at different data split ratios and were evaluated based on their accuracy for classifying precipitation events across all states. Figure 4. illustrates the accuracy achieved by various machine learning algorithms across different states for different train test split ratios.

#### Himachal Pradesh

The analysis revealed that SVC consistently achieved the highest accuracy scores across all train-test split ratios in Himachal Pradesh, ranging from 83.35% to 83.50%. This suggests the effectiveness of SVC in capturing the complex interplay between various atmospheric variables and precipitation patterns in this state. The mountainous terrain and diverse climate of Himachal Pradesh necessitate a robust model capable of handling intricate relationships. RFC followed closely with accuracy scores ranging from 82.84 to 82.94%, demonstrating strong and reliable performance. This indicates the suitability of both SVC and RFC for classifying precipitation in Himachal Pradesh. XGBoost maintained competitive accuracy (82.28−82.75%), while KNN also achieved a reliable score (81.62−81.74%) (Fig. 4). Here, the consistent high accuracy of SVC and RFC suggests their ability to effectively model precipitation patterns in Himachal Pradesh’s diverse topography and climatic zones.

#### Jammu and Kashmir

Similar to Himachal Pradesh, SVC emerged as the most effective algorithm for precipitation classification in Jammu and Kashmir (Fig. 4). The average accuracy across all split ratios for SVC was 79.79%, closely followed by RFC with an average accuracy of 79.61%. This indicates the suitability of both algorithms for capturing precipitation patterns in this state characterized by the Himalayas and Kashmir Valley with its unique weather systems. XGBoost and KNN achieved average accuracies of 78.69% and 77.64%, respectively.

The effectiveness of SVC and RFC in Jammu and Kashmir underscores their ability to handle the complex interactions between atmospheric variables in a region with significant topographic variations.

#### Punjab and Haryana

The analysis revealed SVC as the most effective algorithm across all split ratios for classifying precipitation in Punjab and Haryana (Fig. 4). The average accuracy for SVC was 82.06%, followed closely by XGBoost with an average accuracy of 81.77%. RFC and KNN achieved slightly lower average accuracies of 80.65% and 81.03%, respectively. Interestingly, SVC exhibited superior performance, particularly at higher split ratios, showcasing its robustness in handling varying data distributions. This highlights its suitability for capturing precipitation patterns influenced by diverse factors such as the proximity to the Himalayas and the Indus River basin. The strong performance of SVC in Punjab and Haryana suggests its effectiveness in modeling precipitation patterns in the region’s predominantly flat plains.

#### Rajasthan

In contrast to other states, RFC emerged as the top performer for precipitation classification in Rajasthan. The consistently high accuracy scores achieved by RFC, ranging from 83.55% to 83.71%, indicate its effectiveness in capturing precipitation patterns in this arid state. SVC displayed competitive performance with accuracy scores ranging from 82.34% to 82.79%. XGBoost also showcased consistent performance with accuracy scores between 82.78% and 83.22%. While KNN achieved lower scores (82.00−82.06%), it still offers reasonable predictive capabilities.

#### Uttarakhand

Similar to Himachal Pradesh, SVC emerged as the leader for precipitation classification in Uttarakhand. Its accuracy scores ranged from a high of 84.47% to a low of 84.16% across different split ratios. This consistency suggests SVC’s effectiveness in capturing the intricate relationships between atmospheric variables and precipitation patterns in this state characterized by the Himalayan foothills and diverse microclimates. RFC followed closely with accuracy scores ranging from 83.84 to 83.96%, demonstrating strong performance. XGBoost displayed competitive accuracy (83.33−83.76%), while KNN achieved moderate scores (82.93−83.25%) compared to other ML models.

#### Uttar Pradesh

In Uttar Pradesh, the analysis revealed RFC as the dominant algorithm for precipitation classification. It achieved the highest accuracy scores across all splits, ranging from 84.70% to 85.05%. XGBoost followed closely with accuracy scores ranging from 83.93%  to 84.50%. SVC maintained competitive scores (83.78%−84.16%). KNN achieved moderate accuracy (83.57%−83.73%). The dominance of RFC in Uttar Pradesh, the most populous state in India, can be attributed to its ability to effectively model precipitation patterns influenced by diverse factors such as the Gangetic Plain’s topography and proximity to the Himalayas. The strong performance of both RFC and XGBoost suggests promising avenues for further exploration and potential implementation in operational forecasting systems.

**Fig. 5:**
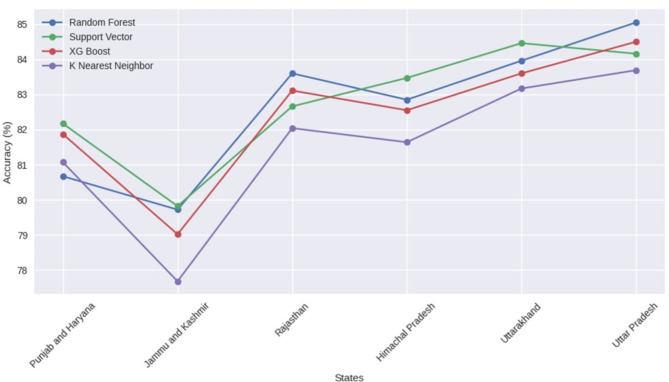
Overall comparison of machine learning performance across the states in North India.

Overall, SVM and RF emerged as strong contenders in our analysis of mapping precipitation patterns across various states in North India (Fig. 5). Notably, SVM consistently outperformed RF in the majority of states, showcasing its effectiveness in accurately classifying precipitation patterns. Interestingly, the RF model exhibited superior performance in Rajasthan and Uttar Pradesh state for precipitation event classification when compared to all the models under consideration. The RF model achieved the highest accuracy scores among all algorithms tested. This observation underscores the nuanced nature of regional climatic patterns and highlights RF’s capability to excel in certain geographical contexts. Upon aggregating the results from multiple iterations and train-test split ratios, we constructed line plot (Fig. 5.) for each state individually, providing a comprehensive overview of algorithm performance.

These plots affirmed the dominance of SVM across most states, but both SVM and RF demonstrated comparable performance, indicating a close competition between the two algorithms. Additionally, the study revealed an inverse correlation between elevation and the performance of machine learning algorithms in simulating precipitation and its extremes over the capital cities in North Indian states. Specifically, in regions with lower elevations, the models exhibited higher skill (accuracy) in simulating precipitation, while in areas with higher elevations, the model skill was relatively lower, with a skill difference of approximately 5%. This observation highlights the influence of topographic features and associated meteorological complexities on the models’ ability to accurately capture precipitation patterns.

### Evaluation of model performance for extreme precipitation

**Fig. 6:**
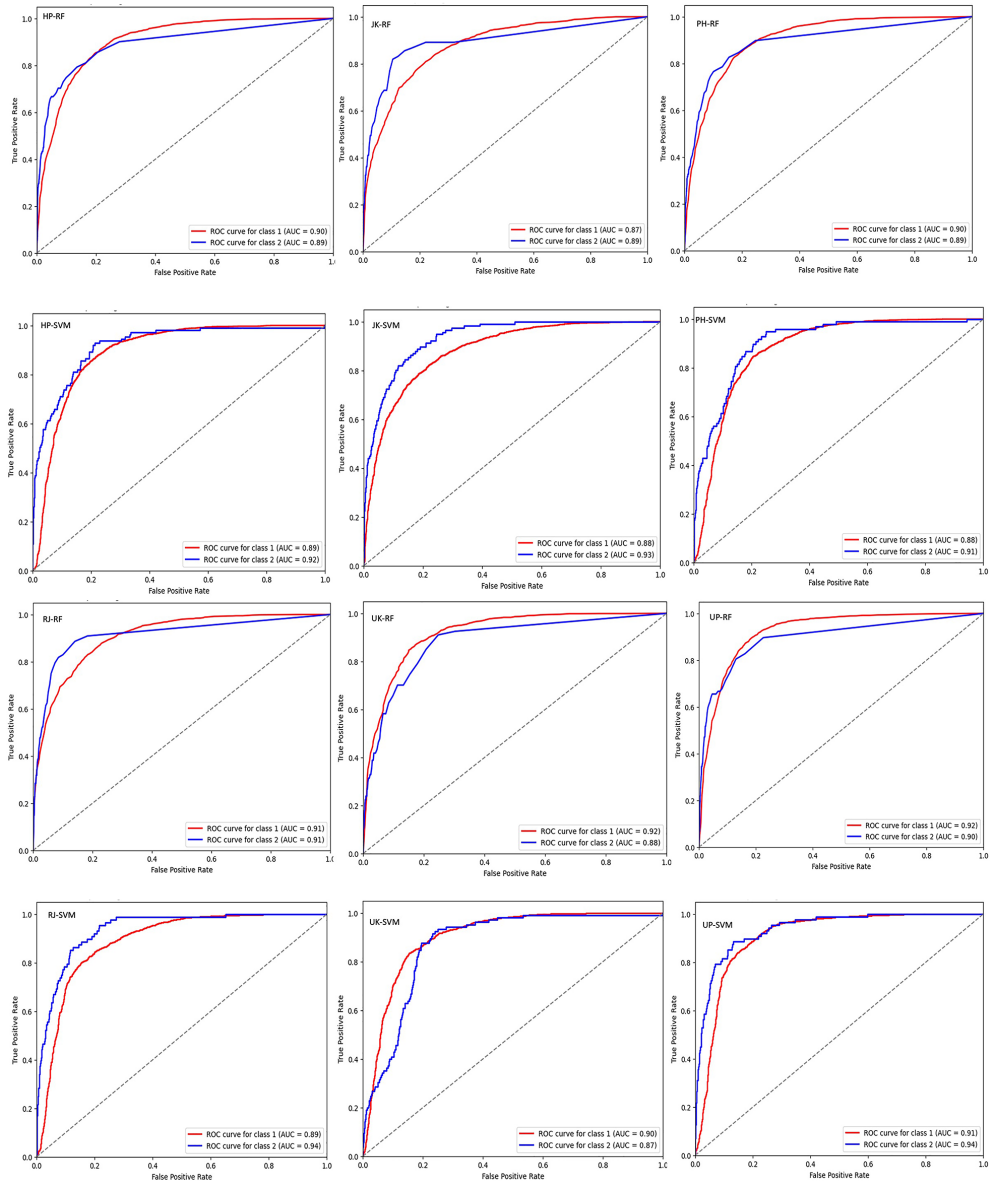
Illustration of ROC curves when all variables are taken into consideration. The illustration showcasing AUC achieved for the 10th percentiles (Class 1) and 95th percentiles (Class 2) in each state. The figures are labelled according to the state initials followed by the model’s name. For instance, HP-RF denotes the Random Forest Model applied to Himachal Pradesh.

**Fig. 7:**
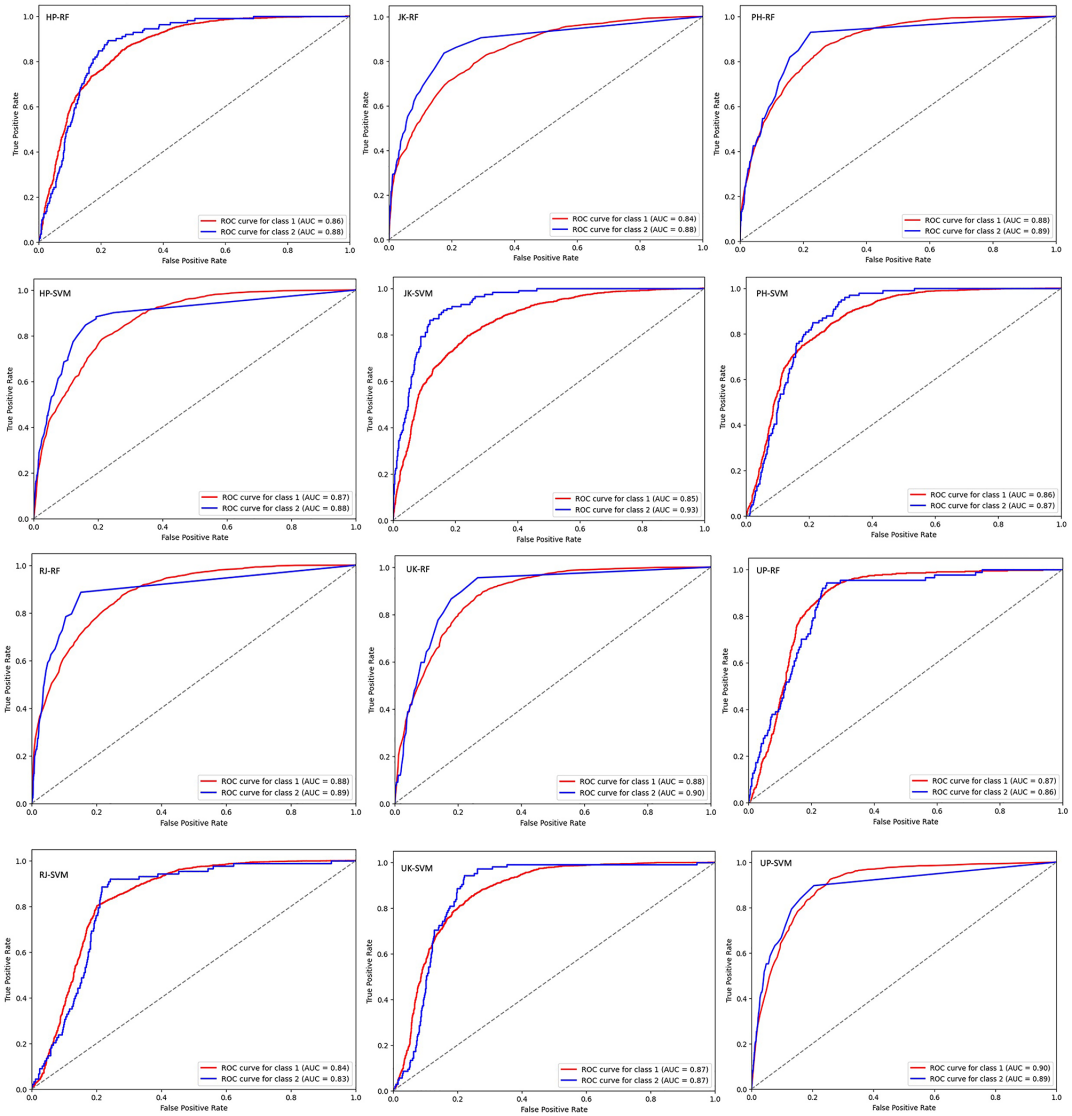
Illustration of ROC curves when statistically significant variables are taken into consideration. The illustration showcasing AUC achieved for the 10th percentiles (Class 1) and 95th percentiles (Class 2) in each state. The figures are labelled according to the state initials followed by the model’s name. For instance, HP-RF denotes the Random Forest Model applied to Himachal Pradesh.

**Fig. 8:**
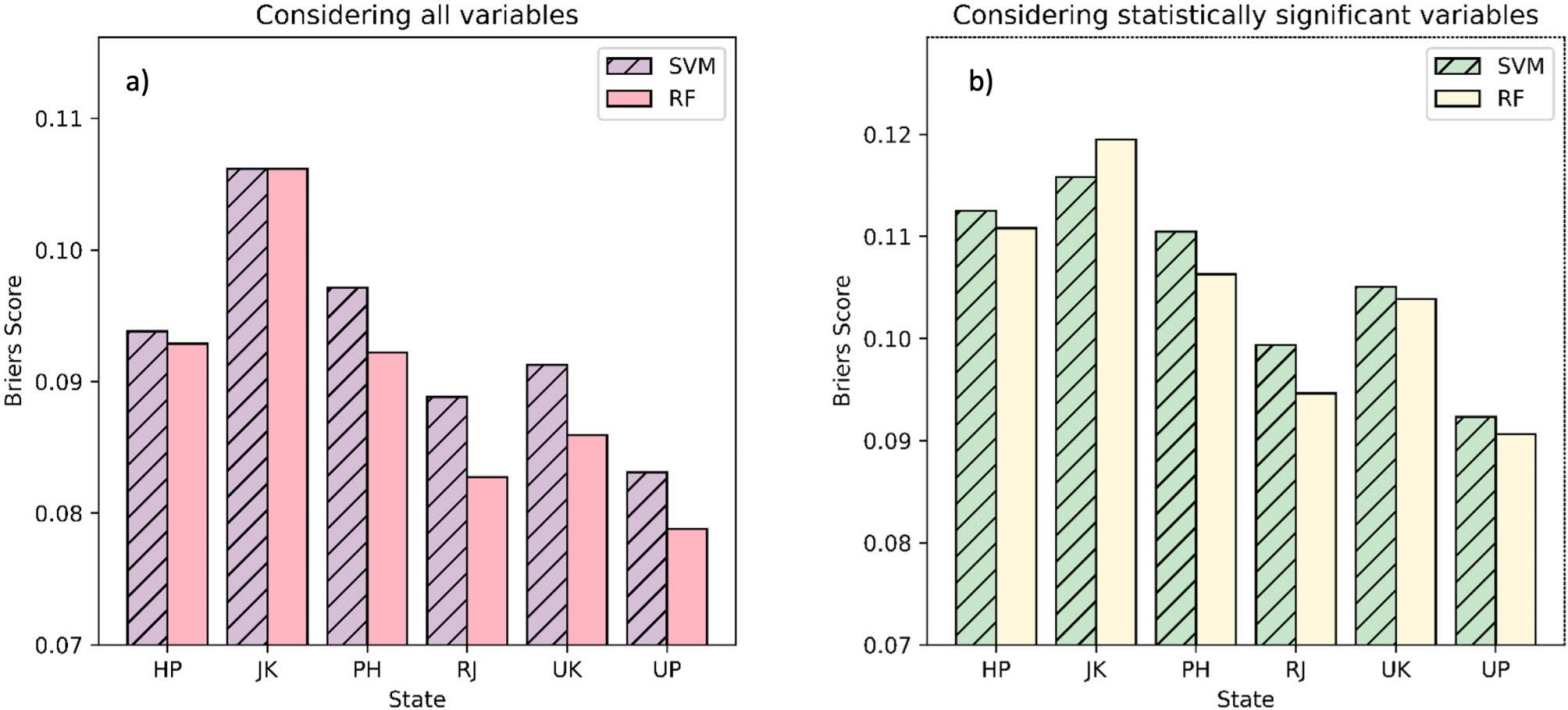
Model Skill Evaluation using Brier’s Score (**a**) considering all variables, (**b**) considering statistically significant variables only.

Building on our previous model evaluation, which demonstrated competitive performance between RFC and SVC models for general precipitation patterns across most states, we ought to specifically evaluate their effectiveness in classifying extreme precipitation events. For this we implemented a two-pronged evaluation for this purpose. First, both models were evaluated using all initial variables (Fig. 6) and then only for statistically significant variables (Fig. 7) across different states. Receiver Operating Characteristic (ROC) curves were generated for each scenario to visualize model discrimination between extreme events below 10th and above 95th percentiles (Figs. 6 and 7). While AUC of an ROC curve provides a good overview of model performance, it’s valuable to consider additional metrics for a more comprehensive evaluation and robustness check of the build models. Here, we considered Brier Score in order to assesses the overall calibration of the model’s classification probabilities with respect to actual outcomes (Fig. 8).

By comparing ROC values and Brier Scores across models and variable selection approaches, we aimed to identify the most effective model for extreme precipitation classification.

- Overall Performance: Both models (RFC and SVC) exhibit good skill in classifying extreme precipitation events, with AUC values generally exceeding 0.85 across states and scenarios (Figs. 6 and 7). This indicates a strong ability to discriminate between extreme and non-extreme events.
- Model Comparison: While there are some variations, the performance of RFC and SVM models is often comparable, with neither model consistently outperforming the other across all states and scenarios.
- Impact of Variable Selection: Focusing on statistically significant variables sometimes results in slightly improved AUC values for the SVM model (e.g., state JK at the 95th percentile threshold) (Fig. 7). However, the differences are generally small, suggesting that the additional variables included in the “all variables” scenario might not significantly impact model performance for extreme event classification.
- State-Specific Variations: There is some variation in model performance across states for the period 1984 to 2023. States like RJ and UP show consistently high AUC values (> 0.90) for both models in both scenarios, indicating exceptional skill in classifying extreme events. Conversely, states like HP and PH show slightly lower but still good performance (AUC around 0.87–0.90). This might be due to topographic differences in extreme precipitation patterns or require further investigation into specific factors or regional dynamics influencing precipitation in those states.
- Threshold Dependence: As expected, AUC values are generally higher for the 95th percentile threshold compared to the 10th percentile threshold. This is because the 95th percentile represents a more extreme precipitation event, which might be easier for the models to predict accurately.

The presented Brier Scores provide additional insights into the performance of Random Forest (RF) and Support Vector Machine (SVM) models for classifying extreme precipitation events across different states. Brier Score measures the mean squared difference between the predicted probability of an extreme event and the actual outcome (0 or 1). A lower Brier Scores indicate better model performance, signifying classification results closer to actual occurrences (Fig. 8).

- Overall Brier Score: The Brier Scores range from approximately 0.07 to 0.12 across states and models. These are generally considered good scores, indicating the models are providing reasonably accurate probability estimates for extreme precipitation events.
- Model Comparison: Similar to the AUC results, there are no significant differences between RF and SVM models in terms of Brier Scores for most states. Both models achieve comparable performance.
- Impact of Variable Selection: Focusing on statistically significant variables (often including humidity and surface pressure) sometimes leads to slightly higher Brier Scores (worse performance) compared to inclusion of all variables. This suggests that while some variables might not be statistically significant, they could still contribute to the model’s ability to calibrate its classification result.
- State-Specific Variations: Brier Scores also show some variation across states. States like RJ and UP consistently have lower Brier Scores (better performance) compared to states like HP and JK. This aligns with the observations from AUC values, suggesting these states might have more predictable extreme precipitation patterns or benefit from the additional information captured by all initial variables.

## Discussion

Extreme precipitation classification using supervised machine learning involves leveraging advanced algorithms to identify and categorize precipitation events based on their intensity and impact. This approach is crucial for improving the prediction and management of extreme weather events, which are becoming more frequent due to climate change. In agreement with previous research, our results demonstrate the efficacy of SVM and RF algorithms in precipitation classification.

Our results on RF’s effectiveness in simulating precipitation extremes in North India are consistent with international studies. Research across the United States^39^ and China^65^ has similarly demonstrated RF’s high accuracy in extreme event classification and forecasting. This global consistency suggests RF possesses inherent advantages for this specific task, possibly due to its capacity to handle high dimensionality and non-linearity efficiently^39,65–67^.

The superior performance of SVM across most states aligns with Nayak’s^68^and vijayakumar’s study^69^ and, who reported its effectiveness in short-term predictions and weather pattern identification. Similarly, RF’s exceptional accuracy in Rajasthan and Uttar Pradesh corroborates^39^ findings on RF’s suitability for regions with distinct seasonal patterns. These consistencies suggest that both algorithms possess inherent advantages in handling the complex, non-linear relationships characteristic of meteorological data.

However, our research diverges from some previous studies in terms of relative algorithm performance. While Shin^70^found RF superior in classifying precipitation types using dual-polarization radar and thermodynamic data, our results indicate SVM superiority in comparison to RF across most of the states, although RF model was concluded as superior model among the two as it depicted low briers score for overall study area. This alignment and discrepancy highlights the importance of considering regional variations and specific data types in algorithm selection for precipitation classification.

The findings partially align with a study conducted in the Pangani River Basin, Northern Tanzania, which similarly identified Random Forest as a top-performing algorithm for extreme rainfall classification. However, while our study found Support Vector Machines (SVM) to outperform Random Forest in most North Indian states, the Tanzanian study reported XGBoost as another top performer, highlighting potential regional variations in algorithm efficacy^71^. Other similar case is with the study over Western Ghats region in India^72^ that showcases the effectiveness of XGBoost and RF models in multi-model ensembles for capturing the behaviour of climate change on precipitation patterns and the success of the improved K-Nearest Neighbor model in simulating such events in New Delhi, India^73^.

Our study’s high accuracy rates, particularly in extreme rainfall classifications, concur with Zhang’ research^74^ who reported true positive rates around 99%. This consistency across diverse geographical contexts underscores the robustness of machine learning approaches in extreme precipitation classification. Furthermore, our findings align with the growing trend of machine learning applications in meteorology, as noted by Abed & Coppola^75^and Hammami & Elasmi^76^, reinforcing the potential of these techniques for improving extreme weather event prediction and climate adaptation strategies.

A novel aspect of our study is the identification of an inverse correlation between elevation and model performance. Models achieved 84% accuracy in lower elevation regions like Uttar Pradesh (123 m), compared to 79% in higher elevation areas such as Jammu and Kashmir (average 1585 m). This 5% discrepancy, attributed to complex orographic effects and potential data scarcity in remote, high-elevation areas, introduces a new dimension to the discourse on extreme precipitation classification. While this finding lacks direct precedent in the literature, it aligns with broader research emphasizing the impact of topographical features on precipitation patterns^72,73^.

Overall, our study largely aligns with existing literature on the efficacy of machine learning in extreme precipitation classification, it also reveals important distinctions in regional algorithm performance and introduces novel considerations regarding elevation’s impact on model accuracy. These findings underscore the need for tailored approaches in different geographical contexts and open new avenues for future research, including the exploration of multi-model ensembles and the integration of temporal modeling techniques such as Long Short-Term Memory networks (LSTMs) to capture sequential patterns in precipitation data.

## Conclusion

In this work, a comprehensive study was conducted to investigate the intricate interplay between atmospheric variables and extreme precipitation events across the seven states in North India. The study leverages combination of data from NASA’s POWER project spanning from 1984 to 2023 and employed four machine learning models (Support Vector Classifier (SVC), Random Forest Classifier (RFC), XGBoost, and K Nearest Neighbors (KNN)) for precipitation classification, specifically for observed extreme event.

The analysis revealed insightful correlations between key predictor variables and extreme precipitation intensity for each region. Dew Point Temperature and Relative Humidity exhibited strong positive correlations (~ 0.4) with precipitation across all states, while Temperature exhibited regional variations with positive correlations in Himachal Pradesh, Punjab and Haryana, and Uttarakhand (~ 0.2), and weaker negative associations in Jammu and Kashmir, Rajasthan, and Uttar Pradesh (− 0.1 to − 0.2). Solar irradiance and Surface Pressure often depicts counterpoints to precipitation, with negative correlations ranging from − 0.18 to − 0.3. The significance of these variables was taken into account while performing probabilistic classification in case of extreme precipitation pattern detection. The predictive classification aspect, employed machine learning algorithms across all states which depicted competitive performance among algorithms.

SVC and RFC emerged as powerful tools for precipitation classification in a changing climate, with SVC dominating in Himachal Pradesh, Jammu and Kashmir, Uttarakhand, and Punjab and Haryana, while RFC excelled in Rajasthan and Uttar Pradesh. The models exhibited higher skill in simulating precipitation over lower elevation regions compared to higher elevation areas, with a skill difference of around 5%, potentially due to the influence of topographic complexity on meteorological phenomena. Furthermore, the analysis of extreme precipitation events revealed that Random Forest models consistently outperformed Support Vector Machines, achieving higher Area Under the Curve (AUC) values (~ 0.90), and lower Brier Scores (~ 0.01), across all states and precipitation thresholds. Despite the promising results, the study acknowledges limitations such as reliance on reanalysis data, limited atmospheric variables, coarse spatial and temporal resolutions and significant effects of climate changes as anthropogenic emission, human induced changes, that were not considered in the present study.

The study’s success with Random Forest models for simulating precipitation extremes among all the models considered in this study, paves the way for further advancements. Future research should explore more advance algorithms like LSTMs and ensemble learning approaches, as well data augmentation strategies and include climate changes factors into consideration. Integrating high-resolution data, climate change projections, and atmospheric processes into the models can enhance their accuracy and robustness. Ultimately, these advancements should be translated into practical tools for real-time flood and drought forecasting, optimizing agricultural practices, and informing adaptation strategies. By considering both atmospheric processes and anthropogenic influences, we can develop comprehensive and holistic models for understanding and both classification and prediction of precipitation extremes, leading to a more sustainable future in the face of climate change.

## Data

The study utilized data from NASA’s Prediction of Worldwide Energy Resources (POWER) project spanning from 1984 to 2023 ^77^ with a daily temporal resolution. The data collection process was centred around obtaining meteorological observations from the capital cities of each state in North India, strategically chosen as representative locations to assess and characterize the atmospheric conditions prevalent across their respective states. This approach aimed to capture the regional variations in climate and weather patterns that influence precipitation dynamics in the region. The study encompassed a comprehensive set of seven atmospheric variables, which were meticulously recorded and analyzed. The atmospheric variables were maximum temperature, relative humidity, surface pressure, wind speed, dew point temperature, precipitation and solar irradiance.

The POWER project provides Meteorological data from Modern-Era Retrospective Analysis for Research and Applications, version 2 (MERRA-2)^77^ and Solar Irradiance data from Clouds and the Earth’s Radiant Energy System Project (CERES). Thus the data was a combination of MERRA-2 ^78–82^ and CERES SYN^83–86^ and has been widely used by researchers for various climate and atmospheric studies. The comprehensive coverage of atmospheric and climate variables, alongside its high spatial and temporal resolution, is underscored by its assimilation of a wide range of satellite observations and other data sources, making it a valuable asset for climate and atmospheric research. MERRA-2 served a grid resolution of 0.5*0.625 degrees and demonstrates advancements in addressing known deficiencies, such as reducing spurious trends and jumps associated with changes in the observing system, as well as mitigating biases and imbalances in aspects of the water cycle^77^. CERES-SYN served a grid resolution of 1*1 degrees. This product provides accurate satellite-retrieved estimates of Earth’s radiation budget components, including downwelling longwave radiation at the surface and other atmospheric levels from 2000 to the present. It utilizes radiation transfer model along with improved cloud property retrievals and consistent temperature/humidity data to estimate radiative fluxes more accurately. As a representative global satellite product covering various timescales, CERES-SYN serves as a valuable data source for evaluating reanalysis radiation estimates over regions lacking sufficient ground observations.

## Methodology

The dataset utilized in this study, has been employed for evaluation of hydrological performance of precipitation products different locations and over Basins^87,88^, for analysis of the diurnal cycle of summer precipitation and associated land-atmosphere interactions^89^, drought estimations^90^ and other hydrometeorological application^91–93^.

The authors used standard setting while performing model simulations to ensure the comparability and replicability of the obtained results (Table 2). We performed feature engineering over the dataset, which include steps as data cleaning, normalization, scaling dropping of any null values present in the dataset.

**Table 2:** Model configuration and workflow.

ML model	Underlying algorithm	Workflow	Parameter setting used for training and evaluation
RF (Random forest)	RF is an ensemble method that uses multiple decision trees to improve classification accuracy. Each tree is built from a random subset of the training data, and the final result is made by averaging the result of all trees^98^.	(1) Collect data (2) Preprocess data (3) Split data into training and testing sets (4) Train multiple decision trees on random subsets of training data (5) Aggregate predictions from all trees (6) Evaluate the model on the test set^37^.	Minmax Scaler, Train-Test Split, Iterations, n_estimators: 100, max_depth: None, criterion: “gini”, random_state: 42
SVM (Support vector machine)	SVM finds the hyperplane that best separates the data into classes. It maximizes the margin between the closest points of the classes (support vectors)^99^.	(1) Collect data (2) Preprocess data (3) Split data into training and testing sets (4) Choose a kernel function (5) Train the SVM model to find the optimal hyperplane (6) Evaluate the model on the test set^37^.	Minmax Scaler, Train-Test Split, Iterations, probability: True, random_state: 42, kernel: ‘rbf’, C: 1.0, gamma: ‘scale’
XGB (XGBoost)	XGBoost is an optimized gradient boosting algorithm that builds an ensemble of trees sequentially. Each tree tries to correct the errors of the previous one^100^.	(1) Collect data (2) Preprocess data (3) Split data into training and testing sets (4) Define the boosting parameters (5) Train the XGBoost model iteratively (6) Evaluate the model on the test set^37^.	Minmax Scaler, Train-Test Split, Iterations, n_estimators: 100, learning_rate: 0.1, max_depth: 6
KNN (K-nearest neighbors)	KNN classifies a data point based on the majority class of its K nearest neighbors in the feature space^101^.	(1) Collect data (2) Preprocess data (3) Split data into training and testing sets (4) Choose the value of K (5) Calculate the distance between the test point and all training points (6) Assign the class based on the majority vote of the K nearest neighbors (7) Evaluate the model on the test set^37^.	Minmax Scaler, Train-Test Split, Iterations, n_neighbors: 5, algorithm: ‘auto’

After the preprocessing, our primary step was to find the intricate relationships between various atmospheric variables. For this, Pearson standard correlations were performed and analyzed^94–97^. The correlation coefficient is a statistical measure often used in studies to represent an association between variables or to evaluate the agreement between two methods. Here, we used it to determine the association between meteorological variables and the target variable ‘precipitation’. The most common measure of correlation can take on values from − 1.0 to 1.0. A value of 1.0 indicates a perfect positive correlation, − 1.0 indicates a perfect negative correlation, and 0.0 indicates no correlation. Generally, Pearson’s correlation between − 0.5 and 0.5 indicates a weak or no association between two variables, while a correlation between 0.5 and 1.0 indicates a strong positive association. In accordance to our data, a value of 0.304 was obtained to be statistically significant at 0.05 significance level. This provided an added insight into the most and least influential parameters affecting precipitation patterns at the selected location.

The next crucial step was the selection and application of machine learning (ML) models to the dataset for precipitation event classification based on atmospheric variables. We employed well-established algorithms like Random Forest (RF), Support Vector Machines (SVM), XGBoost (XGB), and k-Nearest Neighbors (kNN). These models are specifically choosen as they models have consistently demonstrated strong performance in various classification tasks across domains and can capture complex, nonlinear relationships between input features and target variables, which is often the case with environmental and meteorological data. Models like RF and XGBoost are robust to noise, handle high-dimensional feature spaces effectively, and provide feature importance analysis for interpretability^102–107^.

By including tree-based (RF, XGBoost), kernel-based (SVM), and instance-based (kNN) models, we aimed to leverage diverse modeling approaches and capture different perspectives on the underlying relationships between atmospheric variables and precipitation events. Additionally, Table 2 presents an overview of these model working, workflow the model follows and some specific setting for simulations.

The authors employed a comprehensive approach to train models for predicting extreme precipitation events across the North Indian region. The process began with careful consideration of data categorization into two distinct classes to focus specifically on extreme events. Class 1 represented low precipitation or drought conditions (≤ 10th percentile), Class 2 represented extreme high precipitation (≥ 95th percentile), This classification enabled the model to concentrate on both low and high extremes, allowing for a more accurate understanding of the patterns associated with extreme precipitation.

Data splitting strategies was the next step followed after fixing the data classes. Multiple data split ratios were systematically tested to find the most suitable one for balancing training data sufficiency and model generalization. These split ratios included 80 − 20 (80% training, 20% testing), 70 − 30, 60 − 40, and 50–50 splits. The thorough testing of these splits ensured that the selected model could effectively generalize from the training data to unseen testing data, resulting in more reliable predictions.

Model training involved a rigorous process of multiple iterations to optimize performance. Each machine learning model underwent 25 to 30 iterations, with a seed number or random state of 42 used to ensure reproducibility of results. This iterative approach helped fine-tune the models, ensuring better accuracy and efficiency in predicting extreme precipitation events. The combination of well-structured data splits, classification schemes, and iterative training led to the development of robust models capable of effectively identifying and predicting extreme precipitation events in the North Indian region.

**Fig. 9:**
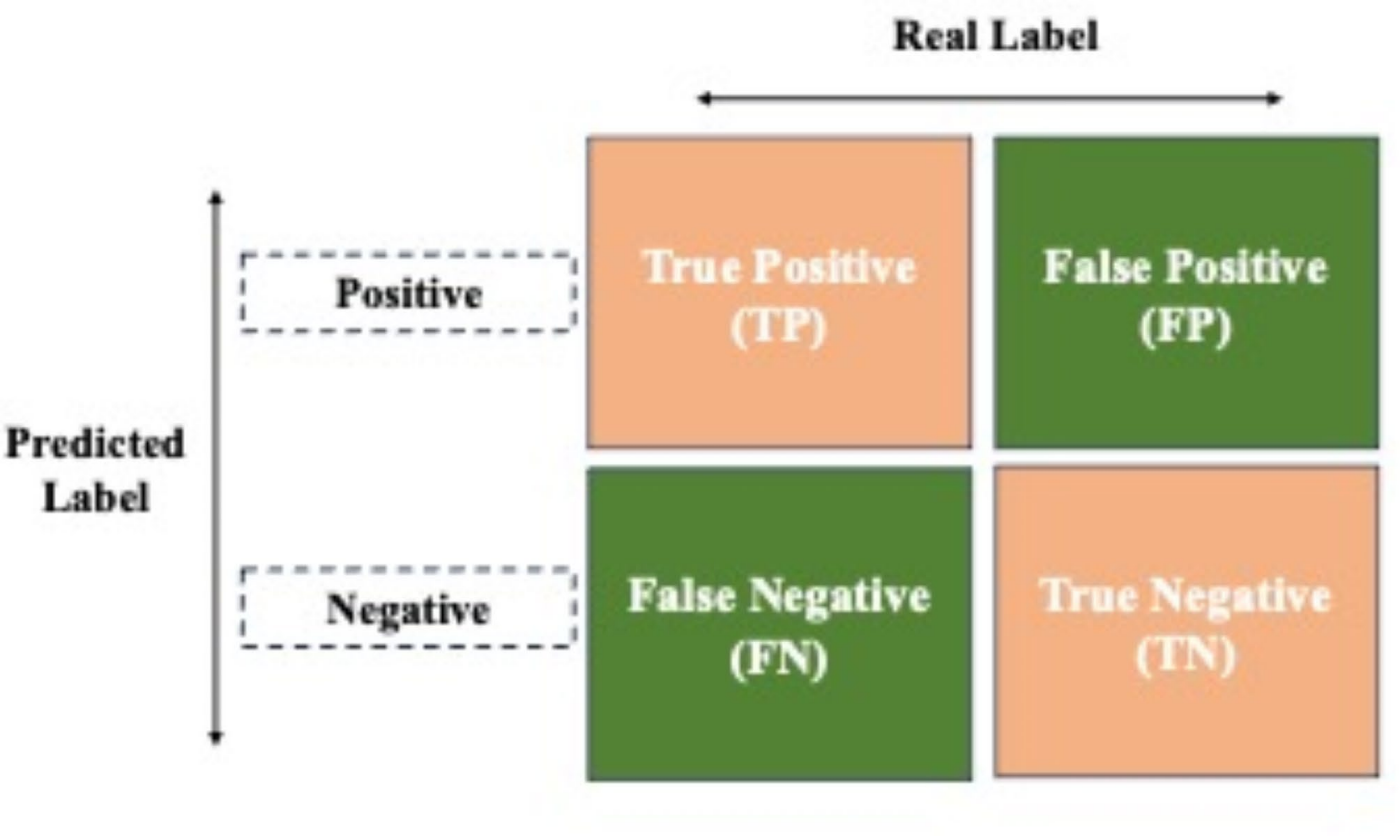
Confusion matrix: a matrix visualization displaying the correct and incorrect classifications made by a model across different classes.

For evaluating our models, we used several metrics as accuracy, sensitivity, specificity, precision, recall, F1-score, and the Area Under the Curve (AUC) and Briers Score. Each metric provides different insights into the model’s performance^94,96,108–110^ and are depicted with their mathematical formulas and definition in Table 3. The calculations are done on the basis of confusion matrix obtained after each iteration. A confusion matrix is a tabular representation that summarizes the performance of a classification model by comparing the predicted class labels with the true class labels (Fig. 9). It provides a comprehensive overview of the model’s accuracy, as well as its tendencies to make different types of errors, such as false positives and false negatives. The rows of the confusion matrix represent the true classes, while the columns represent the predicted classes, and the values in each cell indicate the number of instances that fall into each combination of true and predicted classes. However, we emphasized the selection of ‘accuracy’ as a parameter for forming comparison.

**Table 3:** Evaluation metrics employed in the study, along with their definitions and mathematical expressions.

Evaluation metric	Definition	Mathematical formula
Accuracy	Accuracy measures the ratio of correct predictions over the total number of instances evaluated	$\:\frac{TP+TN}{TP+TN+FP+FN}\text{}$
Precision	Precision measures the positive patterns that are correctly predicted from the total predicted patterns in a positive class	$\:\frac{TP}{TP+FP}$
Recall (sensitivity)	Recall is used to measure the fraction of positive patterns that are correctly classified	$\:\frac{TP}{TP+FN}$
F1 Score	F1-Score is a harmonic mean of precision and recall, providing a balance between the two	$\:2\:\times\:\:\frac{PRECISION\:\times\:RECALL}{PRECISION\:+RECALL}$
Specificity	Specificity measures the fraction of negative patterns that are correctly classified	$\:\frac{TN}{TN+FP}$
Receiver operating characteristic (ROC)	The ROC curve plots sensitivity against the false positive rate at various threshold values	$\:PLOT\:OF\:\left(\frac{TP}{TP+FN}\right)\:vs\:\left(\frac{FP}{FP+TN}\right)$
Area under the curve (AUC)	AUC reflects the overall ranking performance of a classifier	Area under the curve of ROC
Brier’s score	Brier Score measures the mean squared difference between the predicted probability and the actual outcome	$\:\frac{1}{N}\sum\:_{i=1}^{N}{(Fi-Oi)}^{2}$

TP: true positives, TN: true negatives, FP: false positives, FN: false negatives, N: total number of instances evaluated, Fi​: predicted probability for instance i, Oi: actual outcome for instance i (0 or 1)

In order to thoroughly evaluate the performance of each algorithm, with a particular emphasis on their ability to accurately capture extreme precipitation patterns over the North Indian States, we employed a 70:30 train-test split for our data. This specific split ratio was chosen based on established practices in the literature, which suggest that it yields optimal results^111,112^ and also on the comparative performance observed among all splits at the time of accuracy evaluation in the previous segment. To assess model performance across different precipitation intensity levels, we generated Receiver Operating Characteristic (ROC) curves using the 10th and 95th percentile thresholds. These curves not only allowed us to assess sensitivity and specificity but also provided a comprehensive understanding of the Area Under the Curve (AUC), which serves as a valuable metric for quantifying the overall performance of each algorithm in distinguishing between positive and negative precipitation events. The AUC offers a nuanced perspective on the discriminatory power of the models, aiding in the identification of the most adept algorithm for capturing extreme precipitation occurrences in the North Indian region.

In addition to AUC, we also analysed Brier’s Score (Table 3). The Brier score provides a quantitative measure of the accuracy and calibration of probabilistic predictions^108,113^. It is widely used in various fields, including weather forecasting, risk assessment, and machine learning, to evaluate and compare the performance of models that generate probability predictions. A lower Brier Scores indicate better model performance, signifying classification results closer to actual occurrences.

## Data Availability

All the data utilized in this study are openly accessible and can be obtained by contacting the first author, Aayushi Tandon, via email at tdn2408aayushi@gmail.com, or directly from the source: NASA’s POWER Data Access Viewer at https://power.larc.nasa.gov.
